# Three-Appointment Technique to Fabricate Duplicate Denture From Patients Existing Denture

**DOI:** 10.7759/cureus.26094

**Published:** 2022-06-19

**Authors:** Shreya Colvenkar, Santoshi Kumari V, Varalakshmi Reddy, Mohammed Kaleemullah Khan, Akhila Reddy

**Affiliations:** 1 Prosthodontics, MNR Dental College and Hospital, Hyderabad, IND; 2 Prosthodontics, Malla Reddy Institute of Dental Sciences, Hyderabad, IND; 3 Prosthodontics, Malla Reddy Dental College for Women, Hyderabad, IND

**Keywords:** denture, geriatric, satisfaction, flask, duplicate

## Abstract

The duplicating procedure copies most of the features of the existing denture. When a patient gets accustomed to the old dentures, it becomes difficult to adjust to the new set of dentures. That’s the time fabricating duplicate dentures helps. This article describes an easy, cost-effective technique to make duplicate dentures with materials readily available in dental setup. The intaglio surface of the denture was intact which increased patient satisfaction. The reduced vertical dimension was corrected with a new set of teeth. Dentures were delivered to the patient superfast within three days. The patient was totally satisfied with the copy dentures.

## Introduction

The duplicating procedure copies most of the features of existing denture [1-13]. The contour of the original denture preserves the neuromuscular condition of the patient. It is most appropriate when a patient is satisfied with his or her existing dentures but requires a new denture as a backup [3]. This may arise if the denture is broken, lost, or need to be repaired. It also saves the hassle of going through all the steps of denture fabrication. It preserves the function and esthetics without the need to adjust to a new set of dentures [4].

Various techniques of denture duplication have been mentioned in the literature [1-17]. Patients with medical conditions like gastroesophageal reflux disorder experience denture teeth attrition. In such a scenario, the intaglio surface of the denture is usually intact but there is loss of vertical dimension [5]. This article describes a simple method to fabricate a duplicate denture from an existing patient’s denture in only three clinical visits apart from any recalls. It allowed duplication of patient’s dentures intaglio surface together with replacement of new teeth.

## Case presentation

A 78-year-old patient visited the department of prosthodontics with a chief complaint of inability to use her old dentures. During the conversation, she revealed that she had two sets of dentures. The patient was very comfortable with her first set of dentures, but teeth were worn off. This reduced her masticatory efficiency. To overcome that, she made a second set of dentures. The patient couldn’t wear second set of dentures because she couldn’t adapt to the new contours and fit. Her straightforward request was to keep the same denture border and fit as the first dentures and replace only the teeth. She also requested to reduce her visits as much as possible.

First visit

**Figure 1:**
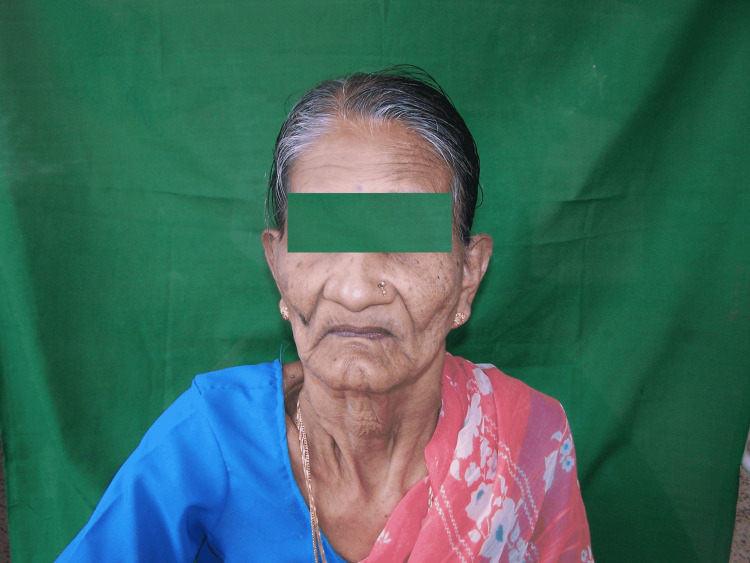
Pretreatment

An oral examination of the patient and the prosthesis was carried out. Retention and stability of dentures were good but the worn occlusal surface of teeth reduced the vertical dimension of occlusion by 3 mm (Figure 1).

**Figure 2:**
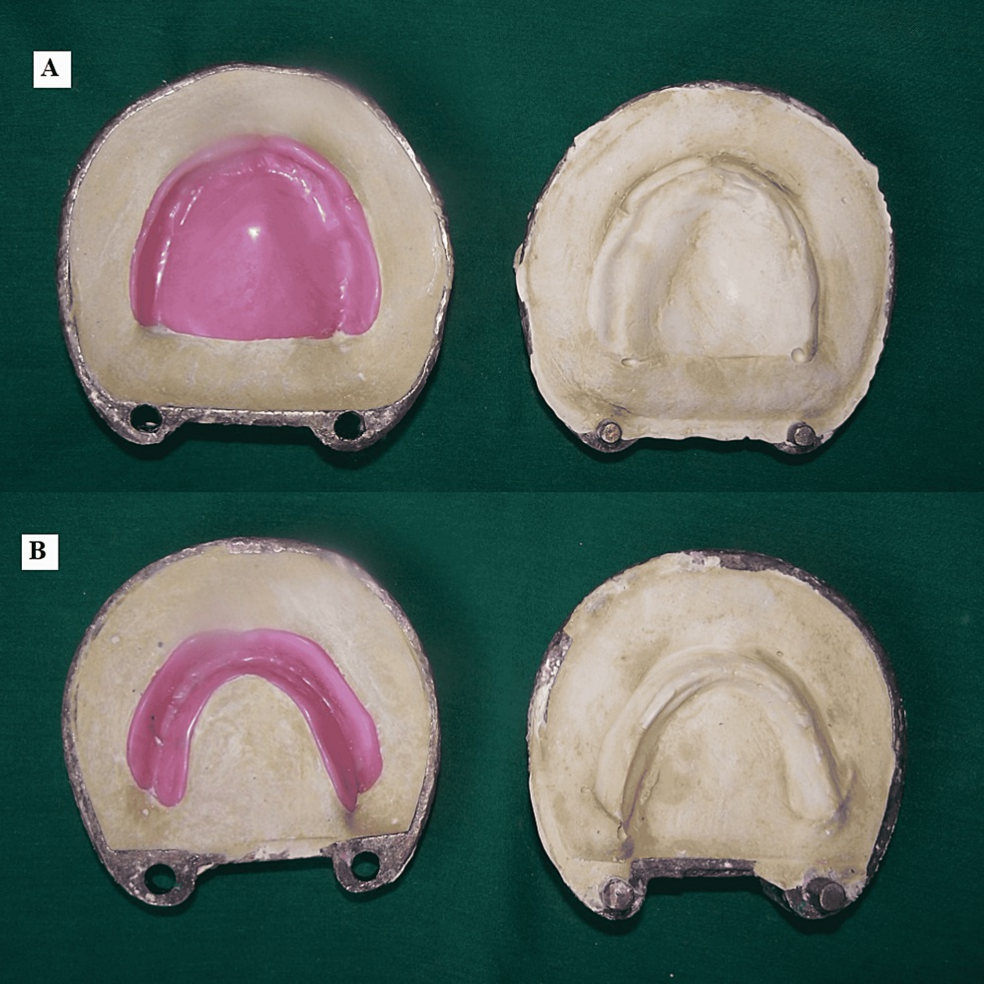
A) Maxillary with second pour of dental stone, B) mandibular denture with second pour of dental stone

There were no undercuts present in the denture. Fabrication of a duplicate denture from the patient’s existing denture was planned. The dentures and the flasks were lubricated with petroleum jelly. Dental stone (Kala Stone, KalaBhai Karson Pvt. Ltd, Mumbai, India) was mixed and poured into one-half of the dental flask. The polished surface of the maxillary denture was embedded into the dental stone (Kala Stone, KalaBhai Karson Pvt. Ltd, Mumbai, India) with denture borders on level with the dental stone. The second section of the flask was placed onto the first section. Petroleum jelly was applied to the intaglio surface of the denture and dental stone (Kala Stone, KalaBhai Karson Pvt. Ltd, Mumbai, India) to easily retrieve the denture. The flask was then filled with the dental stone. The two halves of the flask were separated, and the denture was retrieved once the setting reaction was complete. The same procedure was followed for the mandibular denture (Figure 2).

**Figure 3:**
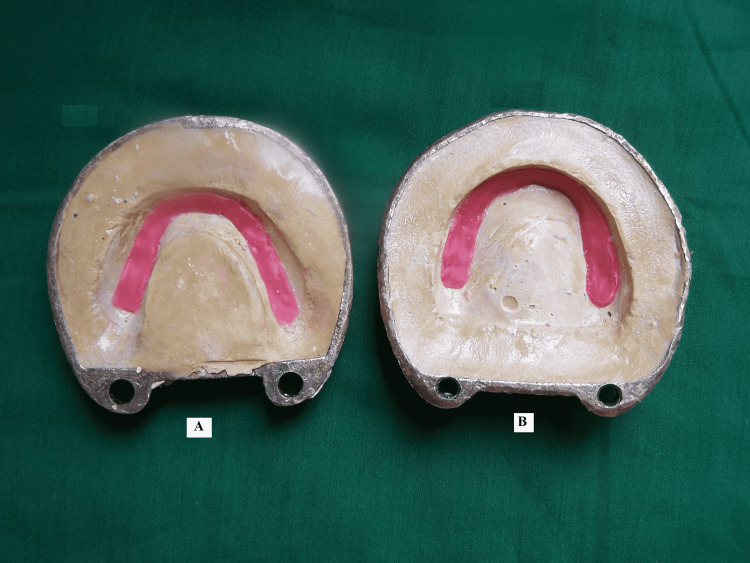
Molten wax poured into teeth impression: A) mandibular; B) maxillary

Petroleum jelly (Vaseline, Hindustan Unilever Pvt. Ltd, India) was applied to impressions of teeth and dental stone (Kala Stone, KalaBhai Karson Pvt. Ltd, Mumbai, India). Molten wax was poured into the impressions of teeth (Figure 3).

**Figure 4:**
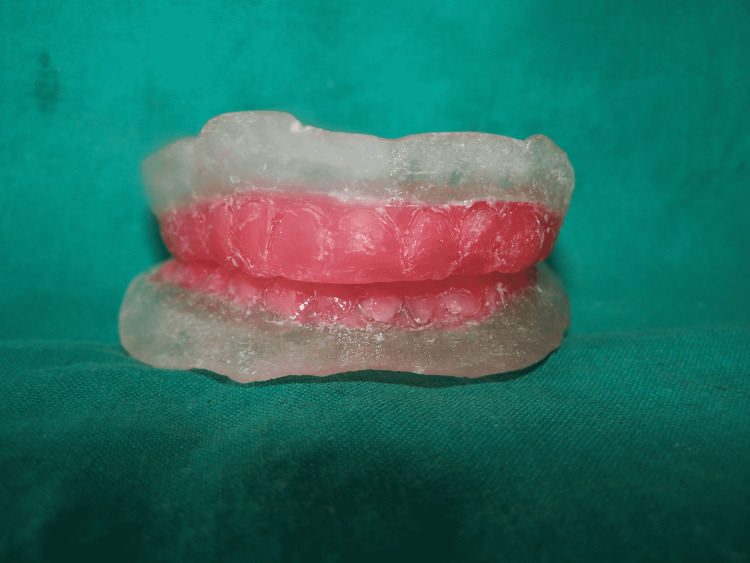
Template dentures

The mold space was then filled with auto-polymerized acrylic resin (DPI RR Cold Cure, Dental Products of India Ltd, Mumbai, India) in dough stage consistency. A separating medium was applied and the second half of the flask was squeezed onto the first. The flasks were secured with an elastic band. The template denture was separated from the flask and excess material was trimmed on completion of polymerization (Figure 4).

As the casts were intact, they were trimmed to the correct size.

**Figure 5:**
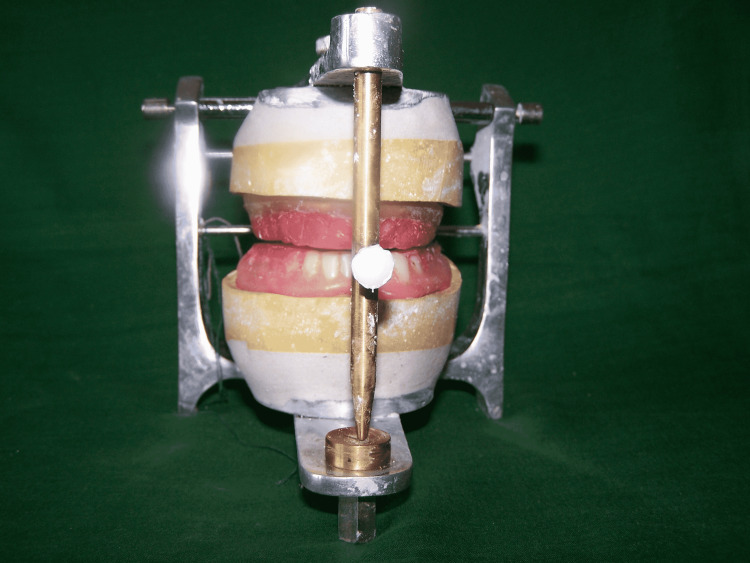
Maxillary template denture with patient’s denture

Jaw relations were recorded by adding dental wax onto the occlusal surface of the denture. The dentures with accurate jaw relations were then mounted on a mean value articulator. The patient’s maxillary denture was removed, and a template denture was inserted (Figure 5).

**Figure 6:**
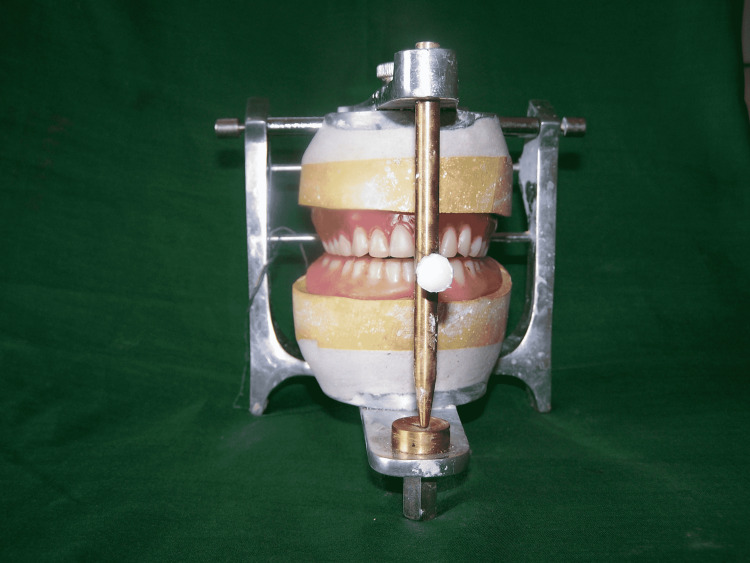
Maxillary denture with patient’s mandibular denture as a guide

The dental wax (DPI Modelling Wax, Dental Products of India Ltd.) was added and adjusted to get a proper occlusal plane. Maxillary teeth setting was completed with patients’ mandibular denture as a guide (Figure 6).

**Figure 7:**
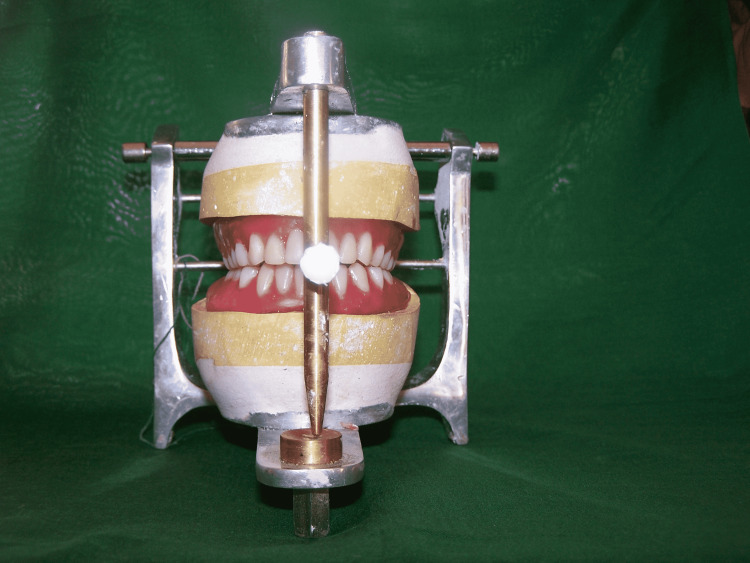
Teeth arrangement

Once maxillary teeth setting was complete, the patient’s mandibular denture was removed, and a trial mandibular denture was inserted. Mandibular teeth arrangement was completed (Figure 7).

Second visit

Once the try-in was accurate, final impressions were made using light body elastomeric impression material (Express, 3M ESPE, St. Paul, MN). The closed mouth technique was followed to maintain accurate tooth relations during the impression procedure. It was then poured into dental stone (Kala Stone, KalaBhai Karson Pvt. Ltd, Mumbai, India). Dentures were processed with heat-cured acrylic resin (DPI Heat Cure, Dental Products of India Ltd.) according to the manufacturer’s instructions.

Third visit

**Figure 8:**
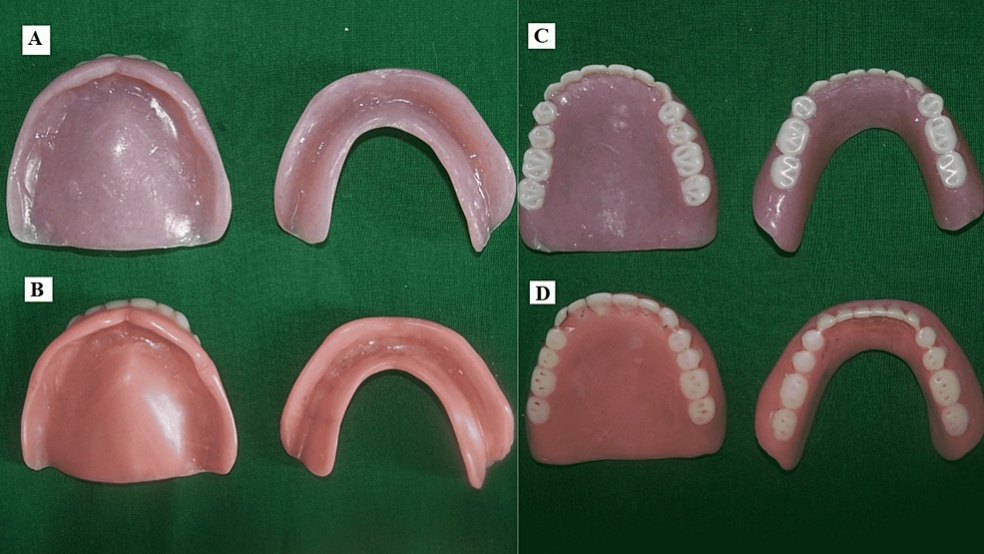
A) Tissue surface of patient’s new dentures, B) tissue surface of patient’s old dentures, C) polished surface of patient’s new dentures, D) polished surface of patient’s old dentures

Dentures with the correct vertical dimension of occlusion and duplicated tissue surface were delivered to the patient (Figure 8).

 

**Figure 9:**
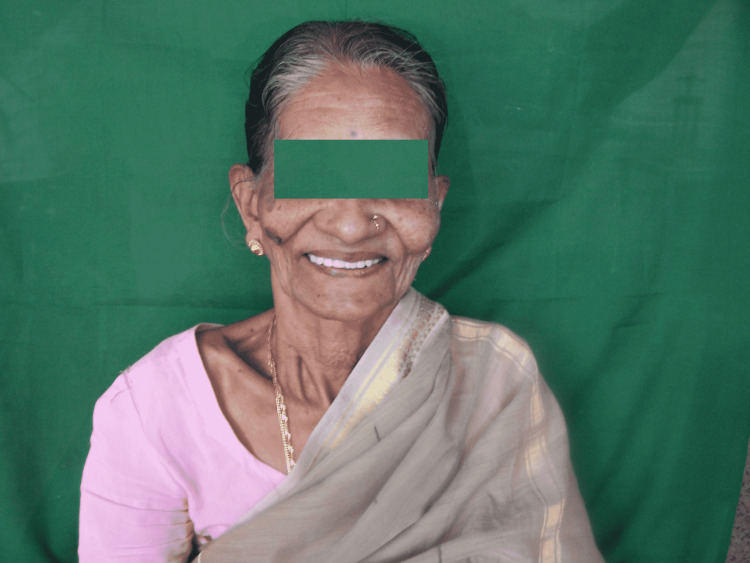
Posttreatment

During recall visit, the patient was satisfied with the function and esthetics of the dentures (Figure 9).

## Discussion

Duplicate dentures were first fabricated in 1960 to be used as spare dentures [1-2]. Getting used to the new fit is a continuous struggle for elderly patients. Dissatisfaction with complete dentures is a known problem and almost 25% of denture wearers have severe problems with their dentures [15-16]. In the present case, the patient was not happy with her two sets of old dentures. It’s very important to do a detailed examination of existing dentures. They provide a great clue in every stage of treatment [17]. The patient was comfortable with her first set of dentures, but teeth were worn off. This reduced her masticatory efficiency. To overcome that, she made a second set of dentures. The patient couldn’t adapt to the new fit of the denture. The patient’s straightforward request was to keep the same denture borders and fit as the first set of dentures and replace only the teeth. She also requested to reduce her visits because of more travel time from her home. She was ready to wait the whole day and complete as many steps as possible. Considering all the factors, duplicate dentures were planned for the patient which would be simple, quick, and less clinically and technically challenging. The patient didn’t have to adjust as the tissue surface was duplicated. It also enhanced neuromuscular coordination, reduced the number of visits, and reduced clinical and laboratory steps.

There are several techniques mentioned in the literature to make duplicate dentures [1-17]. Metal denture flasks are easily available in dental setup to carry out the duplication process. If duplicating flasks are not available, then the trays technique can be used [13].

Irreversible hydrocolloid and silicone putty impression material can be also used as a duplicating material [9,12]. The patient had a specific request about exact duplication of fit as well as denture borders of an old denture, so a wash impression was taken with the patient’s denture. No overextensions were present. During fabrication of the wax template, the cast was totally intact. The same cast was used for mounting the occlusal rims on an articulator.

Patient dentures had worn occlusal surfaces, so nonanatomic teeth were used to prevent denture from getting locked or displaced during function. On the first day, the patient had to wait for a few hours to complete all the clinical steps. On second visit that was the next day, the try-in and wash impression was completed. On third visit, complete dentures were delivered to the patient. The duplicate dentures were processed in three visits instead of five visits normally.

Duplicate dentures provided better esthetics and improved chewing efficiency. The reduced vertical dimension was corrected with the new jaw relation record. Finally, copy dentures incorporated duplicate tissue surface of old dentures and improved chewing function of new dentures.

## Conclusions

For geriatric patients, the copy denture technique is a simple and cheap procedure that duplicates the soft tissue contours. The duplicate tissue surface adds better adaptability to new dentures in terms of function as well as esthetics. In only three visits, dentures were successfully delivered to the patient.
